# Coping Resources of Druze and Bedouin Adolescents During the Iron Swords War

**DOI:** 10.3390/bs16081374

**Published:** 2026-08-10

**Authors:** Fathi Shamma, Dorit Olenik-Shemesh, Tali Heiman

**Affiliations:** 1 Education Department, The Academic Arab College for Education, Haifa 3660801, Israel; 2 Education and Psychology Department, Open University, Raanana 4353701, Israel; doritol@openu.ac.il

**Keywords:** coping resources, Israel, Druze, Bedouin, adolescents, Iron Sword War

## Abstract

This study examined how social and cultural contexts influence coping resources of Druze and Bedouin in the Iron Swords War in Israel. The two Arab minority groups exposed to similar existential threats but with different cultural backgrounds and relations with the Israeli state were studied. A total of 210 adolescents (115 Druze, 95 Bedouin) aged 15–18 years filled out validated questionnaires on sense of coherence, social support, institutional trust, and post-traumatic growth. Findings indicated that compared to Bedouin adolescents, Druze adolescents demonstrated a higher sense of coherence and institutional trust. Family support served as a universal resource for both groups. Bedouin adolescents displayed stronger relationships between external resources and post-traumatic growth, while Druze adolescents seemed to benefit from internal processes of meaning-making. These findings reveal two distinct pathways to post-traumatic growth shaped by structural position: an externally driven pathway for marginalized populations involving resource mobilization against barriers and an internally driven pathway for integrated populations emphasizing meaning-making. The study demonstrates that resilience is not only individual achievement but a social product, requiring both psychological intervention and structural inclusion to support adolescents during wartime.

## 1. Introduction

The world today, as it has always been, is replete with stressors and crises. From the COVID-19 pandemic to the ongoing Russian Ukrainian conflict, from climate change to regional armed conflicts, contemporary society faces multiple overlapping crises that test the resilience of communities worldwide. Several resources have been proposed in the literature on coping during crisis, including individual resources (e.g., emotional, cognitive, and behavioral) as well as community-level resources (institutional organizations and social networks).

While there is significant research on specific coping resources in times of crises regarding how they impact coping and how they are utilized by people during crisis, much less is known about how culture and social contexts may affect individuals’ usage of different resources. This is important because two communities that are confronted with the same threat may use different styles of coping based on their cultural background, social makeup, and identity.

The present study aims to explore how social and cultural contexts shape coping with war in Israel. This study is unique, as it focuses on two Arab minority groups within Israel that are of similar socioeconomic status—middle to lower class—and face the same threat. The two communities compared are Druze and Bedouin Israeli citizens. These two communities are unique, as they are confronted with the same stressor but have very different cultures, social organizations, and relationships with Israeli society.

The central research question guiding this study is as follows: How do social and cultural contexts influence coping strategies employed by middle-to-lower socioeconomic status communities when facing the same existential threat during wartime in Israel? It is essential to draw a clear conceptual distinction between coping strategies and coping resources; coping strategies are cognitive and behavioral actions people enact when drawing on those resources to control and manage stressors (Lazarus & Folkman, 1984). In contrast, coping resources are external and internal resources, such as family and social support, a sense of coherence, and institutional trust, that are accessible and available to individuals and communities (Antonovsky, 1987; Schwarzer, 2024).

The present study focuses on and examines the resources that Druze and Bedouin adolescents possess and the extent to which culture and social contexts shape the access to and use of these resources, rather than measuring the discrete coping behaviors adolescents subsequently employ.

## 2. Theoretical Framework

### 2.1. Transactional Theory of Stress and Coping

The response, adaptation, and coping strategies of a person are determined by the individual’s perception of stress (Walinga, 2010). According to Walinga (2010), “stress has been viewed as a response, a stimulus, and a transaction”. To explain stress as a dynamic process, the transactional theory of stress and coping (TTSC) was developed by Richard Lazarus (Lazarus, 1966; Lazarus & Folkman, 1984). The theory elucidates stress as a dynamic transaction between an individual and his/her environment (Walinga, 2010). The stress theory is a psychological framework designed to elucidate how individuals manage stressful situations (Lazarus & Folkman, 1984). It hypothesizes that to mitigate the detrimental effects of stress on well-being, individuals immerse themselves in different coping strategies.

Biggs et al. (2017) provided an overview of the original and revised versions of the theory. In the original version, emphasis was placed on stress resulting from interactions between individuals and their environment, whereas the revised version emphasized positive emotions (Folkman & Moskowitz, 2004). The main features of the original theory comprise cognitive appraisal and two primary coping strategies: emotion-focused coping (EFC) and problem-focused coping (PFC) (Carver et al., 1989; Folkman & Lazarus, 1980; Lazarus & Folkman, 1984).

The contrastive effectiveness of the PFC and EFC strategies is often the subject of discussion within the coping literature (Biggs et al., 2017). In the revised theory, the main coping strategy is meaning-focused coping (MFC), which entails deriving positive meaning from stressful situations. Thus, stressful situations act as stressors that trigger meaning-focused coping (Folkman, 1997, 2008). MFC evokes positive emotions (such as hope and gratitude), which re-establish resources that impact “cognitive appraisal, sustain coping efforts over time, and provide relief from distress” (Folkman, 2008; Biggs et al., 2017).

### 2.2. Antonovsky’s Salutogenic Approach

Complementing the transactional model, Antonovsky’s salutogenic approach focuses on factors that promote health and well-being rather than solely on pathology (Antonovsky, 1987, 1996). This approach emphasizes individuals’ sense of coherence (SOC) as a key resource for managing stress. Individuals with strong sense of coherence are better able to deal with stressors, as they can comprehend and manage the stressors, as well as feel motivated to face it (meaningfulness) (Antonovsky, 1996). This salutogenic perspective is particularly relevant for understanding resilience and resistance among adolescents in stressful situations.

### 2.3. Post-Traumatic Growth (PTG) Theory

In this study post-traumatic growth (PTG) is a pivotal outcome variable. PTG requires a specific conceptual framework. It refers to positive psychological change experienced as a result of the struggle with highly challenging life circumstances, rather than merely a return to pre-trauma functioning (Tedeschi & Calhoun, 1996, 2004). Tedeschi and Calhoun’s (2004) model conceptualizes growth as emerging through a process in which a traumatic or highly stressful event challenges an individual’s core beliefs and assumptive world, prompting cognitive processing, deliberate rumination, and meaning-making that can, over time, produce positive change across several domains: a greater appreciation of life, more meaningful interpersonal relationships, a greater sense of personal strength, recognition of new possibilities, and spiritual or existential change. Critically, PTG is not the absence of distress; it can coexist with continuing post-traumatic symptoms, as growth and suffering often occur in parallel rather than as opposite endpoints of a single continuum (Tedeschi & Calhoun, 2004).

Within a collective or minority-context framing, PTG is shaped by other individual appraisal processes and by the social and cultural resources available to process the traumatic experience, including family cohesion, community support, and institutional trust (Bar et al., 2025). This positions PTG as a fitting outcome for the present study; inasmuch as Druze and Bedouin adolescents differ in the coping resources available to them, they may also differ in the pathways through which post-traumatic growth is achieved, consistent with the study’s proposed dual-pathway model.

## 3. Literature Review

### 3.1. The Impact of War on Youth

Conflict obviously negatively affects everyone in society; however, war affects youth more than any other group (Vostanis, 2024). Israel is no stranger to war and armed conflict. Most of these conflicts have related to territorial land and issues with identity. Many of these factors involved a complicated mixture between historic, religious, and geopolitical relationships. Some of the recent conflicts that Israel has faced include the Israel–Palestine crisis that escalated in 2021, the Israel–Hamas war that began in 2023, and the ongoing Israel–Hezbollah tension that recently increased in violence in September 2024 (Safdar, 2024; Bordas, 2024; Council on Foreign Relations, 2025; Congressional Research Service, 2024). War conflicts that drag on for years put added pressure on services, economy, jobs, and basic needs, which has a ripple effect on everyone in society (Vostanis, 2024).

A poignant repercussion emanating from warfare that imperils youth well-being is the onslaught of psychological stressors (Solomon et al., 1989; Ward et al., 2021). Adolescents ensconced within war-afflicted vicinities manifest markedly deteriorated mental health profiles, with empirical documentation spanning anxiety disorders, depressive episodes, PTSD manifestations, and compromised social operability (Frounfelker et al., 2020). Studies have consistently documented disparities in mental health among young people exposed to war compared to those in peaceful areas within conflict zones (Attanayake et al., 2009; Slone & Man, 2016).

Israeli minority adolescents face the unique hardship of confronting war. On one hand they must deal with existential fears that all Israeli youth face. On the other hand, they struggle with their ethnic identification in relation to the State of Israel and their place in Israeli society. Understanding how these youth cope (and how much the culture and environment shape their coping) is crucial to creating effective and culturally appropriate programming. This research examines Druze and Bedouin youth, representing two distinct Arab minority communities within Israel. It explores how culture shapes the relationship between outside dangers and individual ways of dealing with them.

### 3.2. The Druze Community

According to CBS (2024), there are approximately 155,000 people in the Druze community. Geographically, they mostly occupy the northern region of Mount Carmel, in Galilee, and on the Golan Heights. The Israeli Druze are descendants of a sect from Ismaili Islam, and their creed assimilates doctrinal elements from a plethora of religious and philosophical lineages. Notably distinct within the Israeli Druze faction is their covenant forged with the State of Israel, established in 1956, mandating conscription into military service for Druze males—a stipulation uniquely exacted from this community amongst Arab citizens of Israel. This draft caused a unique bond with the Israeli government that has been adopted into their story of Druze identity and assimilation. Druze communities have kept tight-knit values of loyalty, pride and unity. They are ruled by a religious government that emphasizes keeping their community secretive and proud (Shamma & Katz, 2018).

### 3.3. The Bedouin Community

Israeli’s Bedouin population approximates 300,000 individuals (Abu Saad, 2023), predominantly located within the Negev Desert’s expanse in southern Israel, alongside minority presences in Galilee. The Bedouin are historically pastoral nomadic Arabs, depending on livestock and farming. Issues concerning the Bedouin have fluctuated between land claims, recognition of villages, and allocation of services. Unlike the Druze community, Bedouin citizens are not conscripted into the military; however, some Bedouin volunteer to serve. Bedouin society is also characterized by strong tribal connections, extended family structures, and cultural values celebrating hospitality and honor. Conflict exists over issues such as land grabs, town planning, and assimilation into Israeli culture versus preservation of their own culture and society. Many Bedouins face marginalization and socioeconomic displacement. Parts of the Bedouin community live in unrecognized villages that lack basic services and infrastructure.

The juxtaposition of these communities offers an unparalleled lens through which the mediation of cultural and social contexts on coping mechanisms amidst wartime can be examined. Both populations share several important characteristics: they are Arab minorities in Israel, they occupy similar middle-to-lower socioeconomic positions, and they face the same external threat during wartime conflicts. On the other hand, they are very different in terms of how they relate to the Israeli state, religious beliefs and practice, communal social organizations, and shared identity. Service in the Israeli military distinctly sets apart Druze Israelis from Bedouin Israelis, who remain marginalized and are engaged in land conflicts. Because these communities have such differences in social and cultural contexts while facing the same external stressors, they provide the perfect opportunity to understand how differing contexts influence the resources and coping mechanisms used by adolescents within each community.

### 3.4. Stress Responses Among Adolescents Exposed to War

A stress situation is the state of a person facing threats to their physical or mental health, due to which they use all their forces and means to protect themself from threats. (Solomon et al., 2005; D’Imperio et al., 2000). Results from other studies have varied since they show conflicting evidence: on one hand, there are different levels of emotional and cognitive symptoms adolescents face when under stress (Laufer & Solomon, 2006; Braun-Lewensohn et al., 2009; Cohen & Eid, 2007; Elbedour et al., 2007; L. Thabet et al., 2007). Another body of research shows that resilience, resistance and even exposure to stress-inducing events can lead to positive outcomes, such as creating social connections and improved self-esteem. These studies indicating resilience and resistance support Antonovsky’s salutogenic approach (Antonovsky, 1987).

The majority of adolescent samples exposed to acute or chronic stressors demonstrated ratings of assorted levels of various emotional symptoms, including psychosomatic symptoms and post-traumatic stress (Laufer & Solomon, 2006; Braun-Lewensohn et al., 2009; Cohen & Eid, 2007; Pat-Horenczyk & Brom, 2007; A. A. Thabet et al., 2009). Research shows that symptoms may appear in both the short and long term, affecting multiple domains: the emotional domain, manifesting in depression and anxiety (Bleich et al., 2006; Elbedour et al., 2007; L. Thabet et al., 2007); the cognitive domain, manifesting in thought confusion and lack of future outlook (Lavi & Solomon, 2005); and the social-behavioral domain, manifesting in withdrawal and internalization.

These stress responses significantly impact adolescents’ daily routine and quality of life, lead to hostility and tendency towards violence and interpersonal conflicts, and impair psychological, familial, social, and academic functioning (Laor et al., 2006).

Research based on the salutogenic approach indicates, however, considerable resilience and resistance among youth (Braun-Lewensohn & Sagy, 2010; Sagy, 2002; Sagy & Braun-Lewensohn, 2009; Disc Zeidner, 2005). Research indicated that stress responses have resulted in positive changes in perceived closeness, trust in social supports, and emotionality between parents/family members (Hobfoll et al., 2006) and positive changes in decision-making skills, optimism, obedience to parents, reliance on family support, and future orientation (A. A. Thabet et al., 2002).

One study found that during the Second Lebanon War, when comparing Arab youth from northern Israel vs. Jewish youth, youth across sectors reported similar and relatively low levels of stress (Braun-Lewensohn et al., 2010). One explanation offered is that in acute war situations, differences between groups become blurred. The literature thus points to diverse emotional and cognitive reactions among teenagers to stressful situations, with some studies emphasizing difficulties and negative symptoms, while others highlight the potential for resilience and growth from crisis (Braun-Lewensohn et al., 2013).

### 3.5. Coping and Minority Stress During the Current Conflict in Israel

Numerous studies have been conducted since October 2023 document coping and psychological distress specifically during the Iron Swords War. This line of research is closely relevant to the current study. Focusing specifically on Bedouin youth, Al-Said and Braun-Lewensohn (2024) examined the coping resources and strategies of Bedouin adolescents during the Iron Swords War and found that personal sense of coherence functioned as a significant protective factor while reliance on nonproductive coping strategies increased distress, with notable gender differences in subjective exposure and emotional reactions. These findings reinforce the relevance of sense of coherence (SOC) and gender as key variables in the present study’s hypotheses.

Beyond adolescent populations, recent work highlights how coping resources operate at the family and community levels during ongoing political violence in Israel. Yeshua-Katz et al. (2025) found that parents residing near conflict-affected areas used WhatsApp groups as a digital coping resource operating across personal, family, and community levels, extending Bronfenbrenner’s ecological framing of coping to online spaces. Bar et al. (2025) further demonstrated, in a study of women exposed to cumulative sexual and conflict-related violence, that personal resources (including SOC and community resilience) mediated the relationship between exposure to violence and post-traumatic symptom severity, underscoring the protective role of the same resource categories examined in the present study.

These studies also point to the importance of minority status as a shaping factor in coping during wartime. Arab minority adolescents in Israel face the compounded burden of coping with a shared existential threat while simultaneously navigating their position as an ethnic and national minority within the state that they, and the adversary, are engaged with (Braun-Lewensohn & Sagy, 2014; Shapira, 2022). This minority-stress dimension distinguishes the coping context of Druze and Bedouin adolescents from that of the Jewish–Israeli majority and motivates the present study’s comparative, culturally situated approach to coping resources.

## 4. Types of Coping Resources

In times of war, the ability to draw support from available resources is essential (Métais et al., 2022). Coping resources are protective factors that can mitigate the impact of crisis and aid recovery from traumatic experiences (Schwarzer, 2024). Coping resources can either be internal or external. Internal coping resources include independence, disposition, mental capacity, self-direction, problem-solving, faith, self-confidence, and communication (Prince-Embury, 2014; Rizzi et al., 2023; Schwarzer, 2024). External coping resources involve family support, social support, community groups, and global assistance (Pak et al., 2023; Schwarzer, 2024).

Resilience is fluid if war victims use both internal and external coping resources (Schwarzer, 2024). Schwarzer (2024) examined two coping resources, self-efficacy and social support, which are internal and external coping resources, respectively. As Schwarzer (2024, p. 6) noted, “in the context of trauma resulting from military conflict, coping resources such as social support and self-efficacy become significant determinants of an individual’s psychological well-being and resilience.” Additionally, psychological coping resources including self-mastery (Stavrova et al., 2020; Hamama et al., 2025) and presence of meaning in life (Hamama et al., 2025; Schnell & Krampe, 2020) have been identified as crucial protective factors.

### 4.1. Personal Coping Resources: Sense of Coherence (SOC)

Individuals with a strong sense of coherence are better able to deal with stressors, as they can comprehend and manage the stressors, as well as feel motivated to face it with meaningfulness (Antonovsky, 1996). Coping strategies can be positively influenced by a high SOC score, which functions as a protective factor (Abreu et al., 2022; Penachiotti et al., 2023). The three components of SOC—comprehensibility, manageability, and meaningfulness—work together to help individuals navigate stressful situations effectively.

### 4.2. Family Support

The role of family in shaping the mental health of youth in situations of war cannot be over-emphasized (Denov & Shevell, 2019). During war, family support includes the provision of coping resources, informing family members (children and adolescents) about current events, and helping family members communicate with deployed loved ones (Pincus et al., 2001; Van Breda, 1999). Furthermore, supporting family members during wars necessitates encouraging those who are discouraged and establishing a sense of trust and security among family members. The family unit serves as the primary buffer against war-related stress for adolescents.

### 4.3. Social Support

Social support is “the subjective feeling of having healthy relationships based on support and trust” (Penachiotti et al., 2023). It can originate from family members, friends, neighbors, colleagues, religious groups, community, organizations, states, and international aid (Lucchetti et al., 2021; Ting et al., 2021; Piankivska, 2022). In times of conflict, there is a need to expand and strengthen networks of relationships, as relying on others for emotional or practical support can significantly impact mental health (Piankivska, 2022). Social support has been considered by researchers as a component of social capital, “which is an investment for access to resources and their future use” (Norris et al., 2008; Piankivska, 2022, p. 77). It has been identified as an important coping strategy to help people cope with traumatic experiences and positively enhance their mental health (Dirkzwager et al., 2003; McKinnon, 2022; Piankivska, 2022).

### 4.4. Trust in Institutions

Trust in institutions has been assessed, revealing that people’s mental health tends to be better when they have positive perceptions toward institutions (Jetten et al., 2020; Žižek, 2020). Trust is necessary for effective public policy delivery by government (OECD, 2017; Skali et al., 2021). According to Galford and Drapeau (2003), “organizations are at particular risk of losing public trust in times of crisis, when feelings of insecurity and perceived lack of safety provoke an accounting of the ways in which, and reasons why, people cannot trust their leaders” (Skali et al., 2021, p. 2). Research by Skali et al. (2021) revealed a lower likelihood of constituents following government voting recommendations during wartimes, highlighting the complex relationship between trust and compliance during crisis.

## 5. Coping Strategies in Response to War

Coping can be defined as the cognitive and behavioral–emotional efforts of an individual to deal with external or internal demands that the individual perceives as burdensome or difficult. Numerous studies have explored the resilience and coping strategies of war victims (Albala & Shapira, 2023; Kimhi et al., 2023; Marciano et al., 2024). Sarid et al. (2025) grouped strategies according to their role in mitigating traumatic effects into self-related (adaptive-coping strategy) and helping others (problem-focused coping).

Al-Said and Braun-Lewensohn (2024) measured coping strategies using the Adolescent Coping Scale and found that problem-solving strategies and nonproductive strategies were significant in determining emotional reactions, consistent with previous studies (Braun-Lewensohn et al., 2009, 2010). Xu et al. (2023), documenting the mental health of Ukrainians during the Russian invasion, reported that productive problem-focused coping strategies “such as active coping and planning as well as emotion-focused coping strategies such as using instrumental support, behavioral disengagement, and self-distraction” were adopted by Ukrainians (p. 964). Wilson and Maceviciute (2025) noted that seeking information related to the source of stress can help alleviate stress and develop effective coping mechanisms.

### Factors Influencing Coping Strategies

While war victims share a collective sense of threat, their responses and coping mechanisms vary considerably. Several factors influence the resilience and coping strategies of youth during war, including age, gender, socioeconomic status, self-mastery, and pre-exposure to traumatic situations.

Sarid et al. (2025) observed that “being female, as opposed to male, and having experienced more traumatic life events predicted higher scores on anxiety, depression, problem focused coping (self), dysfunctional coping (self), and problem-focused coping (other).” Al-Said and Pilpel-Kaiser (2024) also documented differences in coping strategy use between boys and girls, with higher levels of subjective exposures and emotional reactions in females compared to males (Al-Said & Pilpel-Kaiser, 2024; Kheirallah et al., 2022).

Age also influences youth’s responses to psychological distress. Długosz (2023) reported that “younger respondents experienced higher psychological distress” (p. 1). Socioeconomic status further influences coping strategies. Evidence from high-income countries indicates associations between lower levels of depression/anxiety and problem-focused coping and social support seeking, while escape-avoidance is associated with higher depression levels (Taylor, 2007; Matheson et al., 2008). However, such evidence remains unclear in lower–middle-income countries.

## 6. The Present Study

### 6.1. Research Hypothesis

Based on the theoretical framework and literature review, this study tests the following hypotheses:

1. Druze adolescents would report higher levels of sense of coherence (SOC) than Bedouin adolescents during wartime.
2. Bedouin adolescents would report higher family support systems than Druze adolescents during wartime.
3. Druze adolescents would report higher trust in governmental and institutional support than Bedouin adolescents during wartime.
4. There will be differences between boys and girls in terms of the study variables, namely, community resilience, SOC, and coping strategies.

### 6.2. Methodology: Material and Methods

#### 6.2.1. Data-Collection Procedure

Data were collected between April and June 2025; 275 questionnaires were electronically distributed to 30 junior high schools in the north and south of Israel, 210 of which were completed. The questionnaires asked about demographics, subjective exposure, community resilience, SOC, family support and trust institutions. All ethical considerations were followed. As required by the Ministry of Education, a permit issued by the Office of the Chief Scientist was obtained (permit no. 15282). Then, we requested and received the respective principal’s permission to enter the schools. The participation was voluntary and anonymous, and students were free to withdraw their participation during the questionnaire procedure for any reason. The parents sent their consent a week before the completion of the questionnaires. The questionnaire took approximately 20–35 min to complete.

#### 6.2.2. Participants

The sample consisted of 210 participants: 115 Druze adolescents (residing in north Israel) and 95 Bedouin adolescents (residing in south Israel). The age of participants ranged between 15- and 18-years A stratified random sampling procedure was employed to recruit participants for the present study.

Table 1 shows: The sample included adolescents from Druze and Bedouin communities. The Druze group included a balanced gender distribution (48% boys, 52% girls). The Bedouin group had a distribution of (58% girls, 42% boys). Religiosity levels were similar across groups, with most participants identifying as traditional (44% of Druze participants and 43% of Bedouin participants). Participation in youth movements was reported by 41% of the Druze group and 45% of the Bedouin group. The Druze group reported a lower mean age (*M* = 2.29, *SD* = 1.21, *n* = 115) compared to the Bedouin group (*M* = 2.94, *SD* = 1.44, *n* = 95). The overall mean age score for the total sample (*N* = 210) was 2.58 (*SD* = 1.36). Age was recorded using four ordinal categories (15, 16, 17, and 18 years, coded 1–4) rather than as a continuous variable measured in exact years; the means and standard deviations reported above and in subsequent analyses therefore reflect this categorical coding and should be interpreted as an ordinal age index rather than age in years.

#### 6.2.3. Instruments

The study instruments comprised structured and self-reported questionnaires:

- Sense of community coherence: The instrument developed by Antonovsky (1993), as adapted for use across cultural groups by Braun-Lewensohn and Sagy (2011), consists of 13 items on a 5-point Likert scale that explore participants’ perceptions of the world as comprehensible, meaningful, and manageable; in the current study, the scale demonstrated good internal consistency (α = 0.80).
- Social support: The six-item Perceived Social Support scale (Penachiotti et al., 2023), using a 5-point Likert scale, investigated how individuals perceived the backing they received from family, friends, and beyond. Cronbach’s alpha (α) for this questionnaire in the present study was 0.78.
- Trust in relevant institutions and people: The 7-item institutional trust questionnaire (Braun-Lewensohn et al., 2025), adapted and validated specifically within the context of the Iron Swords War among Israeli populations, assessed people’s confidence in the government, the prime minister, the Ministry of Education, the media, social media, and the local council/municipality on a 5-point Likert scale. The questionnaire’s internal consistency in the current study was α = 0.85.
- The state-anxiety measure developed by Spielberger et al. (1970) was used to assess adolescents’ anxiety. This scale consists of 11 items, which are each rated on a 4-point Likert scale. Examples of questions are: “*I feel peaceful*;” “*I am afraid of disasters*;” and “*I am worried*.” The mean score was used, and Cronbach’s alpha reliability coefficient was 0.79.
- State anger was measured using the instrument developed by Spielberger et al. (1970). This scale consists of six items, which are each rated on a 4-point Likert scale (1—*almost never*, 4—*almost always*). Examples of questions include: “*I am angry*;” “*I want to scream at someone*;” and “*I feel frustrated*.” The mean score was used, and Cronbach’s alpha reliability coefficient was 0.75.
- Psychological distress (Ben-Sira, 1979) was evaluated using a five-item psychosomatic symptom scale, in which each item was rated on a 4-point Likert scale (1—*never*, 4—*very frequently*) referring to the frequency of occurrence of familiar symptoms (e.g., headaches, stomachaches). In the present study, Cronbach’s alpha coefficient was 0.82.

## 7. Results

Overall, participants reported moderate to high levels across most study variables. Druze adolescents reported higher scores than Bedouin adolescents on post-traumatic changes, community, support, general feelings, and trust in institutions. The largest difference between the groups was found for trust in institutions. Symptom levels were relatively similar across the two groups.

Table 2 shows: The independent samples *t*-tests revealed significant differences between Druze and Bedouin adolescents across most study variables. Druze adolescents reported significantly higher levels of sense of coherence, community resilience, support, post-traumatic changes, and trust in institutions compared to Bedouin adolescents. The largest difference was found for trust in institutions, with a large effect size (d = 0.83). No significant group differences were found for symptoms. The findings support Hypothesis 1 (Druze adolescents reported higher SOC) and Hypothesis 3 (Druze adolescents reported higher trust in institutions), and Hypothesis 2 was not supported: contrary to the prediction, Druze adolescents reported higher rather than lower family support than Bedouin adolescents. Hypothesis 4 (gender differences) was not tested by this *t*-test, which compared cultural groups only; it is addressed in the MANCOVA reported in Tables 4 and 5 below.

Table 3 shows: Hypothesis 3 predicted that higher levels of trust in governmental and institutional support would be associated with better psychological outcomes more strongly among Druze adolescents than among Bedouin adolescents during wartime. To examine this hypothesis, Pearson correlation analyses were conducted separately for the Druze and Bedouin groups. The findings did not support the hypothesis. Contrary to expectations, trust in institutions demonstrated a stronger positive association with post-traumatic changes among Bedouin adolescents (r = 0.424, *p* < 0.001) than among Druze adolescents (r = 0.298, *p* < 0.001). Similarly, community belonging and social support showed stronger associations with post-traumatic changes in the Bedouin group compared to the Druze group. Among Druze adolescents, however, general feelings as an indicator of sense of coherence demonstrated the strongest correlation with post-traumatic changes (r = 0.586, *p* < 0.001). Overall, the results suggest that institutional trust and related psychosocial resources were more strongly associated with psychological outcomes among Bedouin adolescents than among Druze adolescents. Therefore, Hypothesis 5 was not supported.

Table 4 shows: A 2 × 2 multivariate analyses of covariance (MANCOVA) were conducted to examine the effects of gender and cultural group (Druze vs. Bedouin) on post-traumatic changes, community belonging, general feelings (SOC), social support, and trust in institutions while controlling for age. Preliminary assumption testing indicated a significant Box’s test of equality of covariance matrices, *p* = 0.005. Therefore, Pillai’s Trace was used as the primary multivariate statistic due to its robustness to violations of covariance homogeneity.

The multivariate effect of age was not significant: Pillai’s Trace = 0.016, F (5, 197) = 0.63, *p* = 0.676, and partial η^2^ = 0.016. Significant multivariate effects, however, emerged for gender, with Pillai’s Trace = 0.066, F (5, 197) = 2.78, *p* = 0.019, and partial η^2^ = 0.066, and cultural group, with Pillai’s Trace = 0.165, F (5, 197) = 7.78, *p* < 0.001, and partial η^2^ = 0.165. Importantly, the gender × cultural group interaction effect was also significant, with Pillai’s Trace = 0.057, F (5, 197) = 2.39, *p* = 0.040, and partial η^2^ = 0.057, indicating that gender differences varied across cultural groups.

Table 5 shows: Follow-up univariate ANCOVAs revealed significant interaction effects for community belonging, with F (1, 201) = 4.99, *p* = 0.027, and partial η^2^ = 0.024, and social support, with F (1, 201) = 5.65, *p* = 0.018, and partial η^2^ = 0.027. Specifically, Druze girls reported higher levels of community belonging and social support compared to Druze boys, whereas these gender differences were substantially weaker among Bedouin adolescents.

In addition, significant main effects of cultural group were found across all study variables. Druze adolescents reported significantly higher levels of post-traumatic changes, community belonging, sense of coherence, social support, and trust in institutions compared to Bedouin adolescents.

Regarding Hypothesis 4, the findings partially supported the hypothesis. Significant multivariate gender differences emerged across the combined dependent variables. However, at the univariate level, gender differences were observed only for post-traumatic changes and social support, whereas no significant gender differences were found for community belonging, sense of coherence, or trust in institutions. Furthermore, the significant gender × cultural group interaction suggests that the role of gender differs according to cultural context, particularly in relation to community belonging and social support.

## 8. Discussion

The present study examined coping resources during the Iron Swords War among adolescents in two Arab minority communities in Israel. The Druze and Bedouin face similar existential threats but differ substantially in their institutional relationships, civic integration, and cultural organization. The study follows Lazarus and Folkman’s (1984) transactional theory of stress and coping and Antonovsky’s (1987, 1996) salutogenic model.

Before turning to the interpretation using a hypothesis-by-hypothesis approach, one important limitation should be kept in mind throughout this section. The design is cross-sectional and correlational, and the interpretations offered below, including the proposed distinction between the externally driven and internally driven pathway to post-traumatic growth, describe patterns of differential association rather than established causal mechanisms. These interpretations are theoretically motivated and consistent with the pattern of correlations observed, but they have not been tested directly and should be read as plausible explanations to be evaluated in future research rather than as demonstrated causal pathways. Likewise, where group differences are attributed to specific mechanisms (e.g., military integration, structural marginalization, or possible measurement bias against tribal family structures), these attributions are offered as the most theoretically coherent reading of the pattern of results, not as confirmed explanations; alternative explanations that were not directly measured in this study—most notably, regional differences in the objective intensity and type of war-related exposure between the Druze (north) and Bedouin (south, near Gaza) samples and other unmeasured confounds correlated with a cultural group—cannot be ruled out and may contribute to the observed group differences independently of, or in addition to, the resource-based mechanisms discussed below.

According to the first hypothesis, during wartime Druze adolescents would report a higher sense of coherence (SOC) than Bedouin adolescents. This hypothesis was supported. SOC, according to Antonovsky (1987, 1996), allows individuals to mobilize coping capacities that are available to them in the face of stressors, thus reducing the psychological cost of adversity by rendering it workable and interpretable rather than paralyzing and chaotic.

We can interpret the confirmation of this hypothesis by looking at each community’s position within Israeli society. The Druze maintain a formalized covenant with the Israeli state, whose most visible feature is the mandatory military conscription of Druze males since 1956—unlike any other Arab citizens of Israel (Firro, 1999; Halabi, 2014). Druze adolescents’ collective identity is reinforced through participating in national life, and they experience the community’s role in times of conflict as purposeful and legible. This is communal, so that even threatening events are rendered meaningful. Antonovsky (1996) identified precisely these conditions as determining a strong SOC, and life experiences that are participation-enabling and consistent over time cultivate comprehensibility and meaningfulness.

Bedouin adolescents’ world orientation, on the other hand, is shaped by structural marginalization: the limited state recognition of many Bedouin localities, ongoing land disputes, inadequate public services, and ambiguous civic status (Abu Saad, 2023). When state actions appear exclusionary or arbitrary, and when access to institutional resources is structurally curtailed, the world is less likely to be experienced as manageable. Thus, Bedouin adolescents’ is lower.

According to the second hypothesis, Bedouin adolescents would report higher on family support in relation to Druze adolescents. The reasoning for this was that in the absence of institutional trust and formal civic integration, the family would function as the primary and most accessible buffer against wartime stress. This hypothesis was not supported: Druze adolescents reported that there was significantly higher family support. Because Druze males serve in the Israeli military, the family becomes not merely a background source of emotional support but a coping unit that is actively mobilized, organized around the community’s shared narrative of sacrifice and belonging. Adolescents in such families are likely to experience heightened levels due to emotionally available family support during the conflict period. This is reflected in their higher reported scores.

For Bedouin adolescents, two considerations may help explain why family support was reported at lower levels. First, the Bedouin sample was predominantly female, and adolescent girls may experience family relationships through normative frameworks of gendered obligation, expectation, and behavioral constraint instead of as freely accessible sources of practical assistance and comfort (Al-Said & Pilpel-Kaiser, 2024; Kheirallah et al., 2022).

Second, the measurement instrument used to assess family support may be more sensitive to explicit, verbally expressed, and individually directed family support in more institutionally integrated communities and less sensitive to the collective, obligation-based, and informal forms of solidarity that characterize tribal family structures. This measurement-bias explanation is plausible but remains speculative: it was not directly tested in the present study (e.g., through cognitive interviewing, measurement invariance testing, or a culturally adapted family support measure), and it is offered as one candidate explanation alongside the gendered obligation account above, rather than as an established finding.

The third hypothesis predicted that Druze adolescents would have higher trust in governmental and institutional support. Of all group comparisons in the study, it yielded the largest effect size.

Trust in institutions is a form of macro-level social capital (Norris et al., 2008) that serves during societal crises as a coping resource. When state institutions government, education, military, and local authorities—are perceived by individuals as responsive, legitimate, and acting in their interest, these individuals are more likely to view collective threats as manageable and believe that protection and support will be provided by the broader social order. Research has consistently demonstrated that trust enables more effective cooperation between citizens and state actors in responding to collective threats (OECD, 2017; Skali et al., 2021) and that in crisis conditions positive institutional perceptions are associated with better mental health outcomes (Jetten et al., 2020).

For Druze adolescents, a community history in which the community and the state have been engaged in a relationship of mutual recognition and reciprocal obligation leads to high institutional trust. The state formally acknowledges Druze civic contribution through military integration, and the community internalizes that acknowledgment into a shared identity narrative, thus leading institutions to be experienced as fundamentally legitimate and trustworthy. In wartime, this trust serves as a powerful coping resource, and adolescents’ psychological security is anchored in confidence in the larger social system, not only in personal and familial resources. This resource structure, including individual, communal, and institutional layers, creates a particularly robust mechanism for Druze adolescents facing existential threats.

Bedouin adolescents’ substantially lower institutional trust is produced by a history of adversarial encounters with state authority. These have generated skepticism, ambivalence, and, in many cases, justified distrust toward state institutions (Abu Saad, 2023). During crises, as Galford and Drapeau (2003) observed, institutions face their greatest risk of losing public trust when scrutiny of institutional reliability and intentions is highlighted by uncertainty. Thus, wartime may therefore deepen pre-existing distrust for Bedouin adolescents, rather than the rally-around-the-flag effect that strengthens Druze institutional confidence. The large effect size for this variable underscores that institutional trust is a fundamental dimension of the resources available to adolescents during wartime.

According to the fourth hypothesis, there would be significant gender differences in relation to community resilience, SOC, and coping strategies. The findings provided partial support. What they revealed was a significant multivariate gender effect as well as a theoretically important interaction between gender and cultural group. At the level of individual outcome variables, gender differences were significant specifically for social support and post-traumatic changes, while no significant gender variation was shown for SOC, community belonging, and institutional trust.

Girls reported higher post-traumatic growth, which is consistent with a well-established body of research on gender and coping, according to which female adolescents tend to engage more readily in emotion-processing and meaning-seeking relational coping behaviors (Sarid et al., 2025; Al-Said & Pilpel-Kaiser, 2024). The capacity to reappraise stressful situations and extract positive meaning is a key driver of post-traumatic growth (Folkman, 1997, 2008).

The significant gender × cultural group interaction is possibly the richest finding analytically here, revealing that gender differences are substantially modulated by community membership. Druze adolescent girls reported markedly higher social support and community belonging relative to boys, a gender gap that was considerably attenuated among the Bedouins. Druze girls, less implicated in the direct demands of military participation than the boys and channeled toward relational and community-based resources, are positioned as both recipients and providers of social support, amplifying the gender difference in use of resources.

Among Bedouin adolescents’ social norms, governing female participation in mobility, community life, and access to external social networks may limit the extent to which Bedouin girls can actively mobilize social support and community belonging as formal coping resources, even if they experience genuine solidarity within family and tribal structures. Thus, structural constraints on female participation moderate the expression of gender differences in reported resource use. The broader lesson here is that gender must be analyzed as a variable situated socially and culturally, rather than a universal psychological attribute whose effects across populations are uniform. Interventions targeting adolescent coping during wartime must therefore consider the intersection of gender and cultural contexts and the specific environments within which different groups of adolescents navigate stress and seek resources.

### Limitations

Interpreting these findings requires considering several limitations. First, the convenience sampling of Druze and Bedouin adolescents within each community limits the results’ generalizability to these two minorities. Second, self-report questionnaires were used to collect all data, and these are susceptible to shared-method variance and social desirability bias; future research would profit from triangulating self-report data with teacher-, parent-, or clinician-rated measures. Third, the cross-sectional design does not allow causal inference regarding the direction of the relationships identified, for instance, whether adolescents who have already experienced growth-related meaning-making report greater coherence and trust retrospectively or whether institutional trust and higher sense of coherence promote post-traumatic growth. Panel or longitudinal designs are needed to further identify these pathways.

## 9. Conclusions

This study compared coping resources—sense of coherence, family and social support, institutional trust, community belonging, and post-traumatic changes—among Druze and Bedouin adolescents in Israel during the Iron Swords War. Druze adolescents, whose community maintains a formal covenant with the state including military conscription, reported a higher sense of coherence, family support, community belonging, and institutional trust than Bedouin adolescents from the Negev, whose community’s relationship with the state has historically been more structurally marginalized. Gender differences in social support and community belonging were significant only among Druze adolescents, indicating that gender operates differently across cultural contexts. Taken together, and with the caveats on causal interpretation and unmeasured exposure differences noted above, the findings are consistent with the broader proposition that adolescent coping during wartime is shaped jointly by individual psychological resources and by a community’s structural position vis-vis the state, rather than by either factor alone.

### Practical Recommendations

The findings suggest several directions for practitioners and policymakers who work with adolescents in such communities during periods of conflict. First, strengthening structural links to accessible, trusted institutions may benefit school-based interventions for Bedouin adolescents to partially substitute their lower reported trust in higher-level state institutions. Second, rather than assuming a single model of family support that applies uniformly across cultural groups, interventions should engage existing extended-family and tribal support structures on their own terms. Finally, programming those accounts for local norms around female social participation and mobility is more likely to be effective than an approach that is uniform and gender blind.

## Figures and Tables

**Table 1:** Demographic characteristics of the participants by population group.

Variable	Category	Druze	Bedouin
Gender	Boy	48%	42%
	Girl	52%	58%
Level of Religiosity	Secular	35%	34%
	Traditional	44%	43%
	Religious	21%	23%
Participation in Youth Movement	No	59%	55%
	Yes	41%	45%

Note. Percentages are presented within each population group.

**Table 2:** Independent samples *t*-tests comparing Druze and Bedouin adolescents on the study variables.

Variable	Druze *M* (*SD*)	Bedouin *M* (*SD*)	*t*	*p*	Cohen’s *d*
Post-traumatic changes	4.13 (0.64)	3.79 (0.83)	3.38	<0.001	0.47
Community	3.35 (0.80)	3.09 (0.83)	2.31	0.022	0.32
General feelings (SOC)	3.70 (0.42)	3.48 (0.38)	3.85	<0.001	0.54
Perceived social support	3.82 (0.72)	3.46 (0.86)	3.33	0.001	0.47
Symptoms	2.30 (0.71)	2.13 (0.76)	1.68	0.094	0.24
Trust in Institutions	3.95 (0.94)	3.05 (1.23)	5.99	<0.001	0.83

Note. SOC = sense of coherence. Cohen’s d values of 0.20, 0.50, and 0.80 represent small, medium, and large effect sizes, respectively.

**Table 3:** Pearson correlations for Druze group and Bedouin group.

Variable	1	2	3	4	5
1. Post-traumatic Changes	—	0.152	0.586 **	0.308 **	0.298 **
2. Community	0.344 **	—	0.266 *	0.370 **	0.001
3. General Feelings	0.336 **	0.290 **	—	0.411 **	0.148
4. Support	0.423 **	0.375 **	0.240 *	—	0.002
5. Trust in Institutions	0.424 **	0.438 **	0.230 *	0.278 **	—

Note. Upper triangle: Druze Group (*n* = 115); lower triangle: Bedouin Group (*n* =95). Values below represent two-tailed significance levels (*p*). * *p* < 0.05; ** *p* < 0.001.

**Table 4:** Multivariate analysis of covariance (MANCOVA) examining gender and cultural group differences in study variables while controlling age.

Effect	Pillai’s Trace	F	df	*p*	Partial η^2^
Age	0.016	0.63	5, 197	0.676	0.016
Gender	0.066	2.78	5, 197	0.019 *	0.066
Cultural Group	0.165	7.78	5, 197	<0.001 **	0.165
Gender × Cultural Group	0.057	2.39	5, 197	0.040 *	0.057

The analysis was based on N = 206 complete c. * *p* < 0.05, ** *p* < 0.01.

**Table 5:** Follow-up univariate ANCOVA results.

Dependent Variable	Gender			Cultural Group			Gender × Cultural Group		
	*F*	*p*	η^2^_p_	*F*	*p*	η^2^_p_	*F*	*p*	η^2^_p_
Post-traumatic changes	5.67	0.018	0.027	18.20	<0.001	0.083	2.93	0.089	0.014
Community belonging	4.50	0.035	0.022	1.52	0.219	0.008	4.99	0.027	0.024
General feelings (SOC)	3.34	0.069	0.016	16.43	<0.001	0.076	0.98	0.324	0.005
Social support	4.15	0.043	0.020	16.53	<0.001	0.076	5.65	0.018	0.027
Trust in institutions	0.07	0.798	<0.001	21.40	<0.001	0.096	0.81	0.369	0.004

Note. Age was included as a covariate. Pillai’s Trace was interpreted as the primary multivariate statistic because Box’s test of equality of covariance matrices was significant, *p* = 0.005.

## Data Availability

The data presented in this study are available on request from the corresponding author. The dataset is part of an ongoing research project and is therefore not publicly available.
